# Ferroptosis-Associated Molecular Features to Aid Patient Clinical Prognosis and Therapy Across Human Cancers

**DOI:** 10.3389/fimmu.2022.888757

**Published:** 2022-06-20

**Authors:** Kaisa Cui, Liang Gong, Kang Wang, Yuanben Wang, Liuying Huang, Bingxin Liu, Qilin Li, Qiang Zhang, Bojian Fei, Zhaohui Huang

**Affiliations:** ^1^Wuxi Cancer Institute, Affiliated Hospital of Jiangnan University, Wuxi, China; ^2^Key Laboratory of Carbohydrate Chemistry & Biotechnology, Ministry of Education, School of Biotechnology, Jiangnan University, Wuxi, China; ^3^Department of Radiology, Affiliated Hospital of Jiangnan University, Wuxi, China; ^4^Computer Vision Lab, Department of Electrical Engineering, California Institute of Technology, Pasadena, CA, United States; ^5^Department of Biochemistry, Molecular Cancer Research Center, School of Medicine, Shenzhen Campus of Sun Yat-sen University, Shenzhen, China; ^6^Department of Surgical Oncology, Affiliated Hospital of Jiangnan University, Wuxi, China

**Keywords:** ferroptosis, cancer, survival, tumor microenvironment, cancer therapy

## Abstract

Ferroptosis is a new non-apoptotic form that regulates cell death and is mainly dependent on iron-mediated oxidative damage and subsequent cell membrane damage. Ferroptosis may be a potential therapeutic strategy for immunotherapy, chemotherapy, and radiotherapy in human cancers. Numerous studies have analyzed ferroptosis-correlated signatures or genes, but a systematic landscape of associations among tumor ferroptosis, clinical outcomes, tumor microenvironment, and therapies in human cancers is lacking. Here, we developed a relative ferroptosis level (RFL) combined with drive/suppress regulators and validated it in the Gene Expression Omnibus datasets of ferroptotic drug treatment. Based on this effective evaluation method, we classified about 7,000 tumor samples into high and low RFL groups in each cancer type and observed that high RFL cases demonstrate favorable survival outcomes in nine cancer types from The Cancer Genome Atlas. Then, several RFL-correlated candidate genes that have not been reported to be ferroptosis-related were selected and experimentally validated in five cancer cell lines using Erastin treatment. We further showed that both immunostimulatory and immunosuppressive phenotypes were observed in high RFL tumors, suggesting that the consideration of ferroptosis could be a potential strategy in cancer immunotherapy. Moreover, we found that high RFL cases/cells showed responder or sensitivity to chemotherapy and radiotherapy. Our study provides a comprehensive molecular-level understanding of ferroptosis and may have practical implications for clinical cancer therapies, including immunotherapy, chemotherapy, and radiotherapy.

## Introduction

Drug resistance and low response are great threats to human health and life, though some effective therapies are currently available for cancer. Induced cancer cell death through forms of cell death, such as apoptosis, necrosis, necroptosis, and pyroptosis, has been developed as therapeutic strategies (1). Ferroptosis is a new non-apoptotic biological process for regulating cell death that is mainly dependent on iron-mediated oxidative damage and subsequent cell membrane damage. A series of factors can induce ferroptosis to inhibit tumor growth, such as experimental reagents (Erastin, etc.), anti-cancer drugs (sorafenib, etc.), ionizing radiation, and cytokines (2). For example, a recent study demonstrated that ferroptosis inducers, such as Erastin and sorafenib, activate the AMPK/SREBP1 pathway *via* iron-dependent ferritinophagy, which in turn inhibits BCAT2 transcription, a suppressor of ferroptotic cancer cell death (3). These findings suggest a potential therapeutic strategy for overcoming sorafenib resistance. Moreover, since ferroptosis has been reported to be related to cancer immunity, activation of ferroptosis may serve as a new strategy for developing of anti-cancer agents with immunotherapeutic potential (4). Several recent studies have identified ferroptosis-correlated molecular signatures in multiple cancer types (5–7). Nevertheless, these studies used ferroptosis regulators without distinguishing between drive and suppress regulators. The clinical-related analysis did not reflect ferroptosis levels between high-risk and low-risk groups. Moreover, analysis of immune activity is only performed by immune score using the expression data at the bulk tissue level. Thus, a comprehensive analysis of ferroptosis-associated molecular features in clinical prognosis and therapy across human cancers needs to be explored.

Here, we established a novel relative ferroptosis level (RFL) based on ferroptosis drive and suppress regulators across more than 7,000 cases from The Cancer Genomic Atlas (TCGA) and validated it in the Gene Expression Omnibus (GEO) datasets of ferroptosis drug treatment. We next explored the clinical survival prediction effect of RFL and identified a series of RFL-correlated genes with validation by wet-lab experiments in cancer cell lines. Then, molecular characterization of ferroptosis-associated signatures was analyzed across human cancers. Importantly, the availability of single-cell level data provides an unprecedented opportunity to explore ferroptosis in the tumor microenvironment (TME) in great depth. Moreover, ferroptosis-associated signatures were evaluated for clinically therapeutic liability.

## Materials and Methods

### Data and Resources

Validated ferroptosis driver and suppressor genes were collected from the FerrDb (**Table S1**, http://www.zhounan.org/ferrdb/legacy/operations/download.html) (8). The TCGA RNA-Seq data of primary tumor samples from 21 cancer types were downloaded from the Genomic Data Commons Data Portal (https://portal.gdc.cancer.gov/). Overall survival (OS), age, gender, and race information of each TCGA cancer was obtained from the integrated TCGA Pan-cancer clinical data resource (9). Abbreviations and patient numbers of each TCGA cancer type are included in **Table S2**. Independent datasets GSE104462, GSE121689, GSE32369, GSE132257, GSE103322, GSE123904, GSE87211, GSE83129, GSE34228, GSE16648, GSE81005, and GSE156632 were available on GEO (http://www.ncbi.nlm.nih.gov/geo , **Table S3**). The Clinical Proteomic Tumor Analysis Consortium (CPTAC) CRC and LUAD protein expression data were obtained from https://proteomics.cancer.gov/programs/cptac.

### Identification of RFL-Correlated Genes

According to instruction from FerrDb (8), drivers, which are genes that promote ferroptosis, and suppressors, which are genes that prevent ferroptosis. Then, ferroptosis driver genes and suppressor genes were used to calculate drive and suppress the activity by ssGSEA, respectively. Thus, drive activity represents the level of promoting the ferroptosis biological process and suppress activity represents the level of preventing the ferroptosis biological process. Finally, we defined $\;RFL=\frac{\text{Drive activity}}{\text{Suppress activity}}$ We estimated RFL-correlated genes in the tumors of nine cancers from the TCGA. A total of 19,211 protein-coding genes were selected in the analysis. Those with a correlation between gene expression and RFL of R >|0.3| and *P <*0.05 were considered markedly correlated. The correlation levels were based on the Pearson test. According to a previous study (10), the Jaccard index was used to evaluate the ratio of the RFL-correlated genes common to the two cancer types (10).

### Wet-Lab Experimental Validation of RFL-Correlated Genes

CRC (RKO, HCT116, and HCT8) and LUAD (A549 and H1299) cell lines were cultured as described in previous studies (11, 12). Erastin (MedChemExpress, China) was used to induce ferroptosis in these cancer cell lines. Total RNA was extracted using TRIzol reagent (Takara, Japan). Complementary cDNA was generated using the Prime-Script RT reagent kit (CWBIO, China). The UltraSYBR Mixture (CWBIO, China) was used to detect the relative mRNA expression. The levels of β-actin were used as the reference and normalized control. The primers are listed in **Table S4**.

### Immune Infiltrates Evaluation

Four different methods were used to evaluate immune infiltrates in TCGA tissue-level expression data, namely, ESTIMATE, XCELL, 22 immune cell types (LM22) of CIBERSORTx, and Pan-cancer immunogenomic based on single sample gene set enrichment analysis (ssGSEA) (13–18). The Seurat package in the R software was used to perform single cell cluster and annotation (19). Cells from each patient were annotated as related studies of GSE132257, GSE103322, GSE123904, and GSE156632 (20–22). The Scissor method was used to identify ferroptotic status at single-cell level based on RFL in each TCGA cancer type (23). A TIDE score was calculated online (http://tide.dfci.harvard.edu/) to compute for tumor samples to predict patient response.

### Statistical Analyses, Code Availability, and Visualization

OS, progression-free survival (PFS), and relapse-free survival (RFS) were evaluated by Kaplan–Meier survival analysis and log-rank test as previously described (10, 18). For **Figures 2A–C****,** the clinical samples of each cancer type we used were divided into two groups with high and low RFL group based on the level of RFL. All RFL values from the 10th to 90th percentiles were used as cutoffs to group the samples for survival analyses, respectively; the value yielding the lowest log-rank *P* -value was selected as the cutoff value to group samples. For **Figure 3B****,** the clinical samples of each cancer type we used were divided into two groups with high and low expression based on the expression level of each select gene, respectively. All expression values from the 10th to 90th percentiles were used as cutoffs to group the samples for survival analyses, respectively; the value yielding the lowest log-rank *P* -value was selected as the cutoff value to group samples. Hazard ratio (HR) >1 and *P <* 0.05 were considered significant and associated with poor survival. Hazard ratio (HR) <1 and *P <*0.05 were considered significant and associated with favorable survival. The survival of patients was analyzed by R software 4.1.0 and Graphpad prism 9. Cox regression model analyses were conducted using Graphpad prism 9. T test or Mann–Whitney test was used in the two-group comparison, and the Kruskal–Wallis test was used in the three-group comparison using Graphpad Prism 9. All reported *P*-values were two-sided. Group comparison, receiver operating characteristics (ROC) curve testing, and Chi-squared test analyses were performed using GraphPad Prism 9. Analyses were performed using R software 4.1.0 with the GSVA (1.40.1) package (ssGSEA) for Pan-cancer immunogenomic and enrichment score calculation; survival (3.2–11) package for two-group OS analysis; ESTIMATE (1.0.13) package for ESTIMATE ImmuneScore and TumorPurity estimation; Seurat (4.0.3) package for cluster and annotation in single-cell GC samples; and Scissor (2.0.0) package for identifying of ferroptotic status at single-cell level by integrating bulk sequencing data. A *P-*value of less than 0.05 was regarded as statistically significant. Figures were designed, analyzed, and visualized by GraphPad Prism 9 and R software 4.1.0.

## Results

### Evaluation of Ferroptosis Level by Validated Gene Expression Signatures

**Figure 1:**
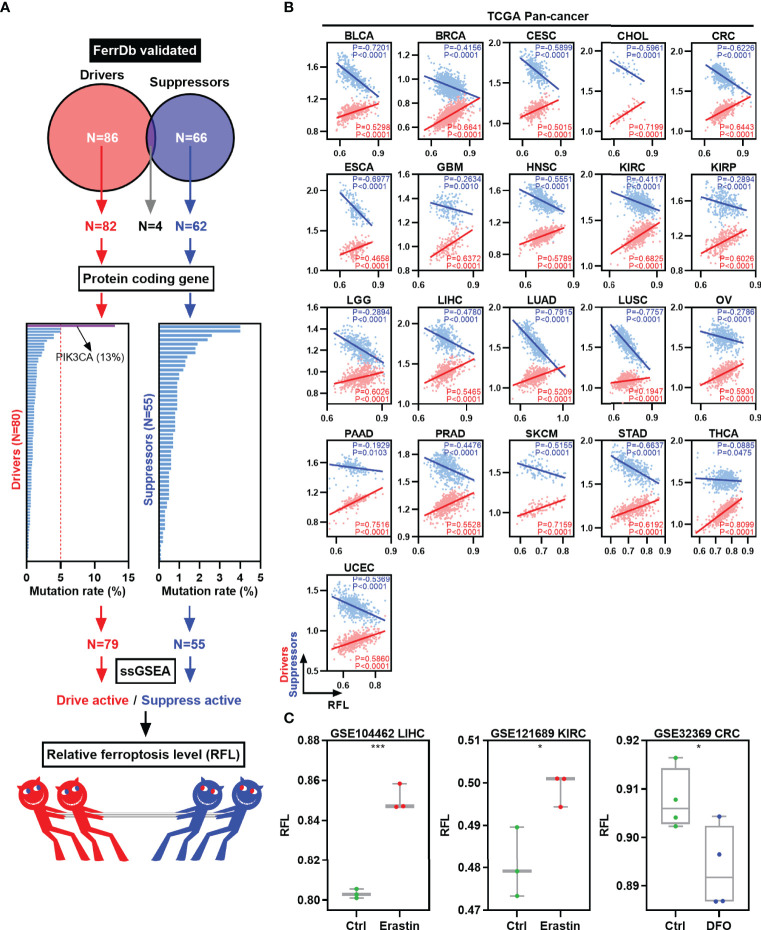
Establishment and validation of RFL in human cancers. **(A)** Graph showing how the RFL was constructed. **(B)** Correlations between RFL and ferroptosis drivers/suppressors active level across human cancers in the TCGA. **(C)** Box plots showing the RFL level in GSE104462, GSE121689, and GSE32369 datasets of cancer cells grouped by Erastin/DFO and control. *P< 0.05, **P < 0.01, ***P <0.001, ****P < 0.0001.

To classify the ferroptosis level of tumor samples, we focused on the FerrDb, the first database of ferroptotic regulator collection in the world (**Figure 1A**) (8). First, validated ferroptosis driver genes (n = 86) and suppressor genes (n = 66) were collected according to the FerrDb. Four genes were excluded because they belong to both the ferroptosis driver and suppressor gene signatures. Next, protein-coding genes were selected for further mutation analysis for reliability. The mutation rates of these genes were lower than 5%, except for PIK3CA (13%). Then, 79 ferroptosis driver genes and 55 suppressor genes were used to calculate the drive and suppress the activity by ssGSEA, respectively. Finally, RFL was established based on drive active and suppressed active.

Multiple analyses were performed to validate the performance and robustness of this RFL. We used the TCGA pan-cancer data to establish RFL and found positive correlations between RFL and drive active and negative correlations between RFL and suppress active in 21 cancer types (**Figure 1B**). Moreover, our data in the GEO datasets showed that cancer cells under ferroptosis inducer Erastin conditions showed significantly higher RFL, whereas those under ferroptosis inhibitor DFO conditions showed significantly lower RFL (**Figure 1C**). These results demonstrate the robustness of the RFL to define and evaluate ferroptosis levels in human cancers.

### High Ferroptosis Level Cases Are Prognostically Favorable in Human Cancers

**Figure 2:**
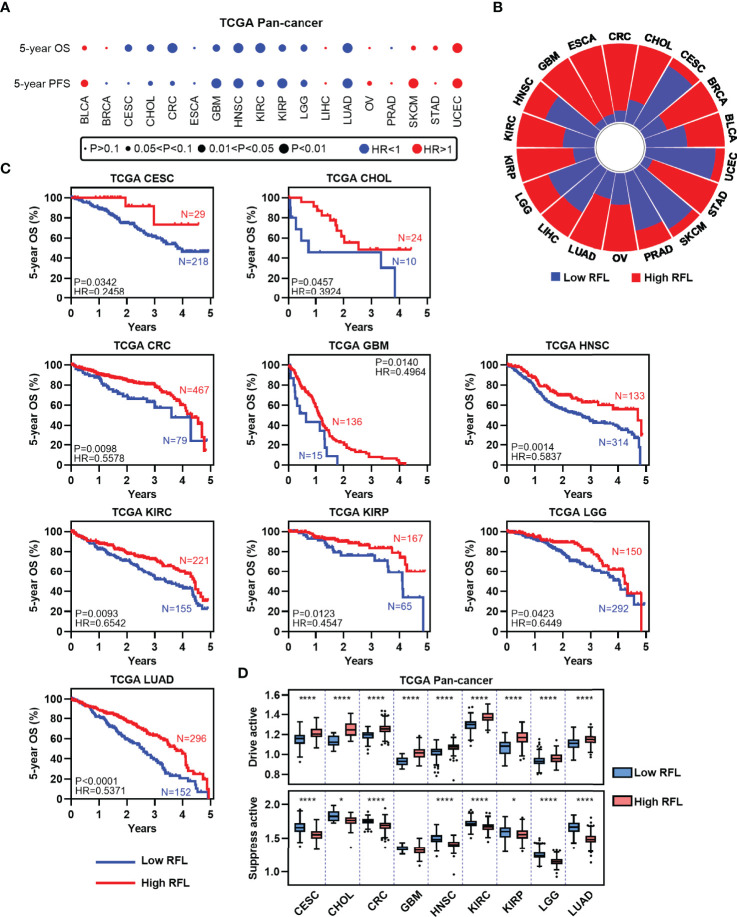
High RFL tumors were consistently associated with worse prognosis across cancer types. **(A)** Association of ferroptotic status with patient OS and PFS times based in different cancer types from the TCGA. **(B)** Percentage of samples with high and low RFL classifications across multiple cancer types from the TCGA. **(C)** Kaplan–Meier plots show that high RFL cases were associated with worse OS time in multiple cancer types from the TCGA. **(D)** Box plots showing the ferroptosis drivers/suppressors active level in TCGA multiple cancer types grouped by **(C)**. *P< 0.05, **P < 0.01, ***P <0.001, ****P < 0.0001.

To assess the relevance of ferroptosis level in cancer clinical trials, we examined the correlations of our RFL classification with the survival time of patients in the TCGA human cancers according to our previous pan-cancer studies (10). LUSC, PAAD, and THCA were excluded with correlations between RFL and drive or suppress active <|0.2|. Interestingly, high FRI predicts favorable OS and PFS in most cancer types (**Figure 2A**). We further concentrated on OS and found that high- and low-RFL groups greatly varied across different cancer types based on OS (**Figure 2B**). Moreover, we observed that high RFL cases were consistently associated with a significantly favorable prognosis across CESC, CHOL, CRC, GBM, HNSC, KIRC, KIRP, LGG, and LUAD (**Figure 2C**). Importantly, potential confounding factors, such as age, gender, race, and TNM stage, were analyzed with RFL to evaluate the independent effect of COX models. High RFL remains a significant independent factor that predicts favorable survival in CHOL, CRC, GBM, HNSC, KIRC, KIRP, LGG, and LUAD, though P = 0.0557 in CESC (**Supplementary Figure 1**). Additionally, the high RFL group demonstrated higher ferroptosis drive active in these cancer types, whereas the low RFL group demonstrated lower ferroptosis suppress active (**Figure 2D**). Hence, these results suggest the potential prognostic power of RFL classification, which considers ferroptosis drive and suppress regulators.

### RFL-Correlated Genes Identified by RFL Predict Patient Outcome in Human Cancers

**Figure 3:**
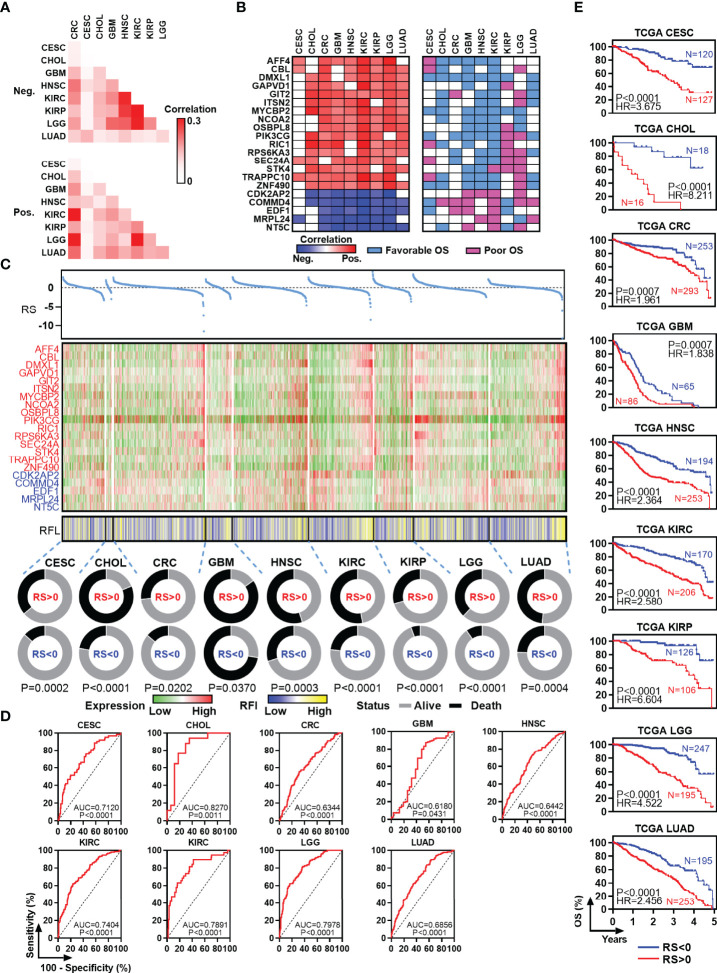
Identification and survival analysis of RFL-correlated genes. **(A)** Heatmap visualizing the matrix of Jaccard indices of the shared connections for the negative upregulated (top) and positive (bottom) RFL-correlated genes of each cancer from the TCGA. **(B)** Heatmaps showing the correlation level between RFL-correlated genes and RFL (left), as well as survival type (right). **(C)** Multivariate COX regression models showing the effect to OS of RS based on 21 RFL-correlated genes in TCGA cohorts. Dot plot shows the risk score (top). Heatmaps showing the 21 RFL-correlated genes and RFL levels (middle). Pie charts show the OS status in RS <0 and RS >0 groups (bottom). **(D)** ROC plots showing the AUC of survival based on the RS in TCGA cohorts. **(E)** Kaplan–Meier plots show that high RS cases were associated with poor OS time in multiple cancer types from the TCGA.

To identify new RFL-correlated genes in human cancer, we analyzed correlations between RFL and protein code genes across CESC, CHOL, CRC, GBM, HNSC, KIRC, KIRP, LGG, and LUAD. A series of RFL-positive and -negative genes have been identified in these cancer types, and we used Jaccard indexes to evaluate the overlap of these RFL-correlated genes in each cancer type. RFL-positive genes showed different patterns than RFL-negative genes (**Figure 3A**). Next, we found 21 RFL-correlated genes were positively (n = 16) or negatively (n = 5) correlated with RFL in at least seven cancer types (**Figure 3B**). Interestingly, most of these RFL-positive genes exhibit favorable OS in human cancers, while RFL-negative genes show poor survival. These genes were further selected to develop Cox regression models for each cancer type (**Figure 3C**). The risk scores decreased as the RFL and RFL-positive genes were expressied, while they increased as RFL-negative genes were expressed in most cancer types, except LGG. The high-risk group (risk score of >0) had more death status compared with the low-risk group (risk score of <0). Moreover, our analysis showed that there was a significant difference in prognosis and poor survival between the high and low-risk groups in CESC, CHOL, CRC, GBM, HNSC, KIRC, KIRP, LGG, and LUAD (**Figures 3D, E**). These data hint that the models according to the RFL-correlated genes identified by RFL are effective in the prognosis of human cancers.

**Figure 4:**
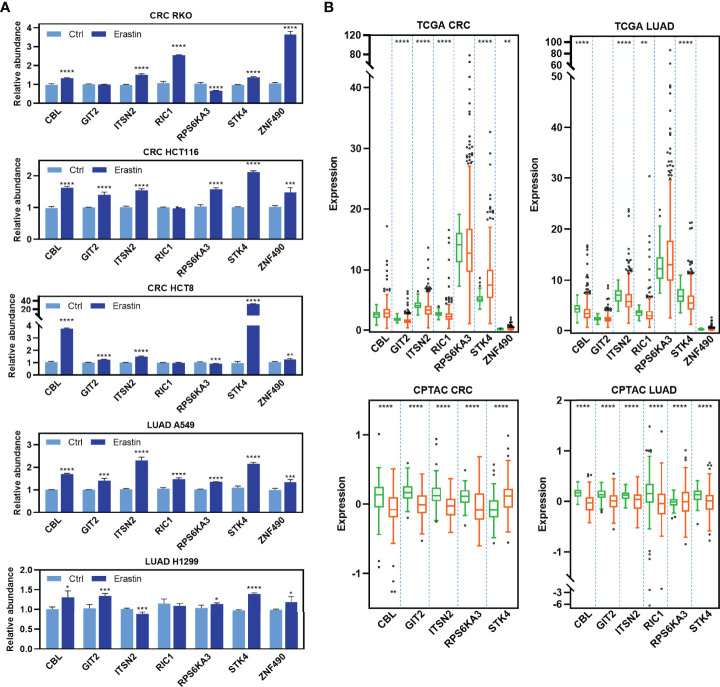
Functional validation of RFL-correlated genes. **(A)** Barplots showing expression levels of seven candidate RFL-correlated genes after Erastin treatment in five cancer cell lines. **(B)** Box plots showing expression levels of seven candidate RFL-correlated genes grouped by tumor and normal samples in the TCGA (mRNA) and CPTAC (protein) cohorts. *P< 0.05, **P < 0.01, ***P <0.001, ****P < 0.0001.

To determine the functional significance of the RFL-correlated genes identified by RFL above, seven candidate genes that have not been reported to be ferroptosis-related were selected and experimentally validated in five cancer cell lines. Most of these genes were upregulated after Erastin-induced ferroptosis in RKO, HCT116, HCT8, A549, and H1299 cell lines (**Figure 4A** ). Intriguingly, most of these RFL-positive genes were downregulated in tumor tissues at mRNA and protein levels of CRC and LUAD (**Figure 4B**). Thus, RFL-correlated genes identified by RFL are associated with Erastin-induced ferroptosis and tumorigenesis. Our findings suggest the potential of the RFL, which can be an effective resource to functionally identify RFL-correlated genes.

### Correlations Between Ferroptosis-Associated Signature and TCGA Molecular Subtypes Across Cancer Types

The above mentioned data demonstrate robustness, potential prognostic, and identify the power of the RFL. We therefore explore the molecular characteristics of different RFL groups based on previous subtype studies performed by the TCGA Research Network, etc. The RFL high group comprised more MSI/CpG island methylator phenotype (CIMP) tumors in CRC (**Supplementary Figure 2A**). The MSI is mostly located in the right colon and is frequently associated with the CIMP (24). Interestingly, current clinical dogma considers MSI tumors to carry a good prognosis and a low risk of relapse (25). Previous research has exhibited a sustained clinical response to immune checkpoints (ICs) with dramatic clinical improvement in CRC patients with MSI-H (26). In HNSC, the RFL high group showed fewer classical cases compared with the RFL low group (**Supplementary Figure 2B**). TP53 mutation, alteration of oxidative stress genes, and heavy smoking history occurred in most classical tumors (27). RFL high group showed more proximal-inflammatory subtype and lower proximal-proliferative subtype in LUAD (**Supplementary Figure 2C**) (28). In KIRC, high RFL cases comprised more m1 and m3 subtypes, while RFL low cases had more m2 and m4 subtypes (**Supplementary Figure 2D**) (29). High RFL KIRP patients showed a few C2c subtype cases compared with the low RFL group (**Supplementary Figure 2E**). C2c tumors consisting solely of CIMP-associated KIRP patients, who were younger at the time of presentation and had a lower probability of OS, were also characterized by a Warburg-like metabolic shift to glycolysis-dependent metabolism and upregulation of hypoxia-related genes (30). In LGG, the low RFL group showed a higher proportion of IDH wild-type patients (**Supplementary Figure 2F**). The large majority of LGG without an IDH mutation had genomic aberrations and clinical behavior strikingly similar to those found in primary GBM (31). In GBM, high RFL cases largely overlap with the mesenchymal subtype, which showed high levels of genes in the tumor necrosis factor superfamily pathway and NF-κB pathway, suggesting that higher overall necrosis and associated inflammatory infiltrate (**Supplementary Figure 2G**) (32, 33). These data provide an overview of the molecular differences associated with RFL across human cancers, particularly parts of characterization of these molecular subtypes that are associated with RFL level and matched clinical outcomes.

### High Ferroptosis Level Cases Demonstrate Immunostimulatory Ground

**Figure 5:**
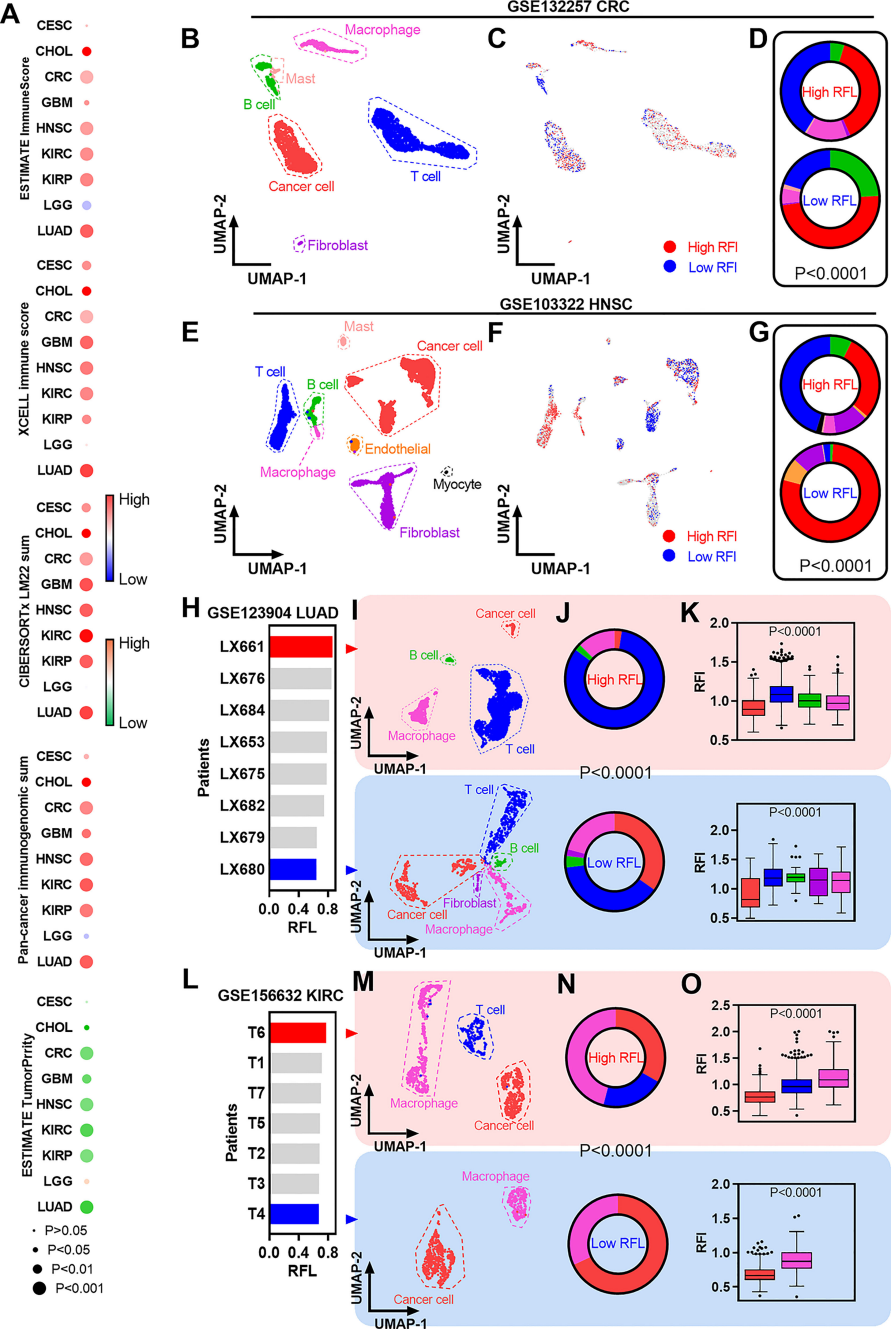
Immune characterization of ferroptosis-associated signatures across cancer types. **(A)** Heatmaps showing the correlation levels between RFL and total immune infiltration/tumor purity. **(B)** UMAP plot showing cell type clusters in the GSE132257 dataset. **(C)** UMAP plot showing high and low RFL cell subpopulations in GSE132257 dataset. **(D)** Pie charts showing the distribution of cell types in high- and low-RFL cell subpopulations related to **(B,C)**. **(E–G)** Simila to **(B**–**D)**, but in GSE103322 dataset. **(H)** Barplots showing the RFL in patients from the GSE123904 dataset based on pseudo bulk transcriptome data. **(I)** UMAP plot showing cell type clusters in LX661 and LX661 patients from the GSE123904 dataset. **(J)** Pie charts showing the distribution of cell types in LX661 and LX661 patients. **(K)** Box plots show RFL levels grouped by cell types in LX661 and LX661 **(L–O)** Similar to **(H–K)**,but in GSE156632 dataset.

Analyses of the molecular characterization of ferroptosis showed that high RFL was associated with inflammatory infiltrates. Recent studies have suggested correlations between ferroptosis-correlated factors and proinflammatory TME, which activates anti-tumor immune responses, suggesting a unique relationship between ferroptosis and TME (34, 35). To further investigate this, four independent methods were employed to evaluate immune infiltrates in CESC, CHOL, CRC, GBM, HNSC, KIRC, KIRP, LGG, and LUAD (13–17). Notably, RFL was positively correlated with total immune infiltrates and negatively correlated with tumor purity in these cancer types from TCGA, except LGG (**Figure 5A**).

The availability of single-cell transcriptomic profiles across a broad range of human cancers provides an unprecedented opportunity to explore TME in greater depth. Thus, we used single-cell datasets from GEO to gain a better understanding of ferroptosis-correlated TME. First, fresh cell preparation single-cell data of CRC were used to analyze and we identified cancer cells and a series of nonmalignant cells (**Figure 5B**). New biotechnology that identifies cell subpopulations from single-cell data according to a given phenotype-associated bulk expression data,was used to identify high- and low-RFL cell subpopulations in this CRC single-cell data (**Figure 5C**) (23). Interestingly, the high-RFL cell subpopulation contained more nonmalignant cell types (i.e., T cells and macrophages) and fewer cancer cells compared with the low-RFL cell subpopulation (**Figure 5D**). We also performed similar analyses on HNSC single-cell data and observed more significant results, suggesting that high-RFL has a comparatively fertile ground of immune cell infiltration in TME (**Figures 5E, G**). Next, we used another method to analyze LUAD single-cell data, and patients with primary tumors were selected. The mean values of single-cell expression in each patient sample for each gene were calculated as pseudobulk transcriptome data to obtain RFL at the tissue level of each case (**Figure 5H**). The LX661 and LX680 patients showed the highest and lowest RFL, respectively (**Figure 5I**). Then, we identified cancer cells and a series of nonmalignant cells in these two samples. Impressively, the high-RFL (LX661) sample exhibited more T-cell infiltration and lower tumor purity compared with the low-RFL (LX680) sample (**Figure 5J**). Furthermore, RFL was downregulated in cancer cells compared with nonmalignant cell types (**Figure 5K**). We also analyzed the KIRC single-cell data of primary tumors and observed similar results (**Figures 5L–O**). These results hint that RFL reflects immune cell infiltration and tumor purity in the TME of human cancers. The level of ferroptosis should be considered and could be a potential strategy to provide immunostimulatory in human cancers. This strategy has been reported in a new study of nanomedicine, which demonstrates simultaneous targeting of both the TME and cancer cells (36).

### Ferroptosis-Associated Molecular Signature in Clinically Therapeutic Liability

**Figure 6:**
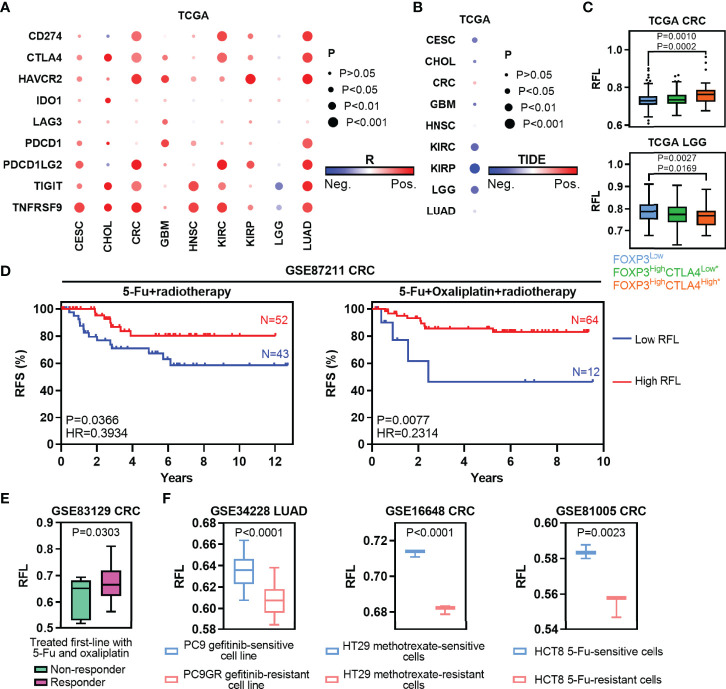
Ferroptosis-associated molecular signature in clinically therapeutic liability. **(A)** Heatmaps show the correlation levels between RFL and IC expression. **(B)** Heatmaps show the correlation levels between RFL and TIDE values. **(C)** Box plots showing RFL levels grouped by each FOXP3/CTLA4 subtype in TCGA cohorts. **(D)** Kaplan–Meier plots show that high RFL cases were associated with a higher risk of relapse after chemotherapy/radiotherapy in the GSE87211 dataset. **(E)** Box plots showing the RFL level grouped by non-responder and responder after being treated first-line with 5-FU and oxaliplatin in the GSE83129 dataset. **(F)** Barplots showing RFL levels grouped by sensitive and resistant after treated gefitinib, methotrexate or 5-FU in GSE34228, GSE16648, and GSE81005, respectively.

To characterize the clinically applicable therapeutic implications of RFL, we first examined cancer immunotherapy due to the abovementioned data of TME. Immunomodulators play a critical role in cancer immunotherapy and we found that most of the immunomodulator genes were overexpressed in high RFL tumors, except LGG (**Supplementary Figure 3**) (37). Next, nine ICs were selected according to the previous TME study, and RFL was positively correlated with these IC expressions in CESC, CHOL, CRC, GBM, HNSC, KIRC, KIRP, and LUAD from the TCGA, except LGG (**Figure 6A**) (38). These results suggest that high RFL is correlated with an immunosuppressive phenotype. Besides, RFL was negatively correlated with the TIDE score, which is computed for tumor samples and can serve as a surrogate biomarker to predict the response to ICs blockade (**Figure 6B**). The patient who have low TIDE might represent the response to IC inhibitor-based treatment (39). However, CRC showed a weak positive correlation between RFL and TIDE. These data on TME, ICs and response prediction motivated our next analysis in immune overdrive subtype, a novel TME-based immune molecular subtyping system have reported in previous research (18). The FOXP3^High^CTLA4^High*^ subpopulation (Treg subpopulation with overexpressed CTLA4) was characterized by an immune overdrive phenotype with high immune cell infiltration, low tumor purity, high IC levels, and TGF-β activation in CRC, and observed in LGG. The FOXP3^High^CTLA4^High*^ cases showed higher RFL compared with other groups in the CRC (**Figure 6C**). Nevertheless, we noted that the FOXP3^High^CTLA4^High*^ cases have the lowest RFL compared with other groups in LGG, consistent with the abovementioned data. These results suggest that patients with tumors have a relatively higher ferroptosis level, which may be related to combination treatment with immunotherapy, particularly IC inhibitor combination treatment.

Traditional chemotherapy or radiotherapy remains the selection of first-line therapy in multiple cancer types. Thus, we first evaluated the effect of ferroptosis status in preoperative radiochemotherapy of CRC. High RFL was associated with a lower risk of recurrence in CRC patients with no matter 5-FU + radiotherapy or 5-FU + oxaliplatin + radiotherapy (**Figure 6D**). Responder cases treated first-line with 5-FU and oxaliplatin demonstrated higher RFL than non-responder cases (**Figure 6E**). Chemotherapeutic drug-sensitive cancer cell lines, such as gefitinib (PC9), methotrexate (HT29) or 5-FU (HCT8), show a higher RFL compared with matched drug-resistant cell lines (**Figure 6F**). Taken together, our analyses show that the ferroptosis status meaningfully correlated with tumor response to immunotherapy, chemotherapy, or radiotherapy. Thus, the tumor ferroptosis status should be considered to improve the efficacy of these cancer therapies.

## Discussion

**Figure 7:**
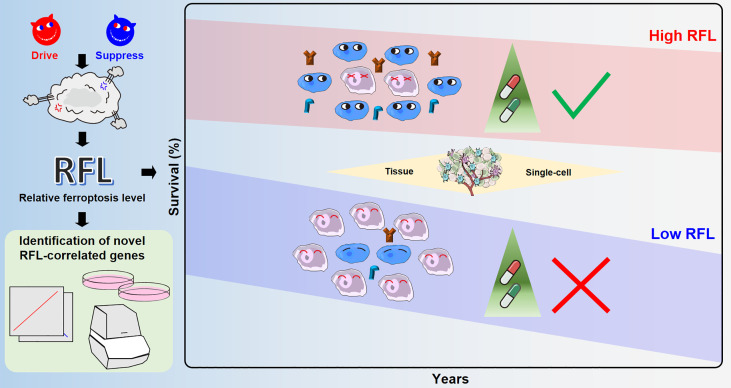
Overview of ferroptosis-associated molecular features to aid patient clinical prognosis and therapy across human cancers.

Ferroptosis induces cell death that influences tumorigenesis and is associated with a series of cancer therapies. Therefore, understanding the characterization and effect of ferroptosis on molecular signatures is crucial to providing new strategies for cancer therapies. Previous studies have analyzed ferroptosis-correlated signatures or genes in single-types of cancer, but we noted that a systematic landscape of associations among tumor ferroptosis, clinical outcomes, tumor microenvironment, and therapies in human cancers is lacking. Besides, recent studies have also analyzed ferroptosis-correlated molecular signatures and constructed scores for these cancer types (5–7). However, most of these studies analyzed ferroptosis signatures without distinguishing between drive and suppress regulators. Different risk groups based on RFL-correlated genes also lacked reflection of ferroptosis relative level. Thus, we constructed a novel relative ferroptosis level (RFL) based on the ferroptosis drive and suppressors across human cancers. Validated ferroptosis driver and suppressor genes collected from FerrDb were used in this study. Drivers are genes that promote ferroptosis, and suppressors are genes that prevent ferroptosis (8). Then, ferroptosis driver and suppressor genes were used to calculate the drive and suppress the activity by ssGSEA, respectively. Thus, driving activity represents the level of promoting the ferroptosis biological process, and suppressing activity represents the level of preventing the ferroptosis biological process. Then, we defined $\text{RFL}=\frac{\text{Drive activity}}{\mathrm{suppress activity}}$ . This index was used to classify tumor cases into high- and low-RFL groups in each cancer type to examine the survival prediction effect in each cancer type, as well as adopt the COX model to evaluate independent effectiveness with potential confounders, including age, gender, race, and TNM stage. Notably, patients who showed a high ferroptosis level tended to have better survival outcomes in CESC, CHOL, CRC, GBM, HNSC, KIRC, KIRP, LGG, and LUAD, while lower RFL cases showed poorer survival (**Figure 2** and **Figure S1**). Cancer cells under the Erastin-induced conditions showed significantly higher RFL, while DFO-induced conditions showed lower RFL (**Figure 1** ). These results suggest that RFL could simulate and reflect ferroptosis levels in tumors. Furthermore, genes identified by RFL could predict survival and are associated with the ferroptosis-level alternation in cancers (**Figures 3**, **4** ). Thus, ferroptosis levels could help evaluate the clinical outcomes and identify new targets of ferroptosis across human cancers. Furthermore, our integrative analyses further demonstrated an overall positive relationship among ferroptosis, immune features, and response to cancer therapies using RFL (**Figures 5** – **7** ).

We noted that RFL was always less than one in tumor tissues or cancer cell lines of related datasets used in this study, such as TCGA tumor tissues (**Figures 1B**, **6C**), GSE87211 CRC tissues (**Figure 6D**), LIHC/CRC/LIRC cancer cell lines (**Figures 1C, 6C**), and cancer cells in single-cell data (**Figures 5K, O**). Moreover, although nonmalignant cell types showed higher RFL, which is more than one or slightly less than one, RFL based on pseudobulk transcriptome tumor expression levels in the single-cell data is still less than one, even if the proportion of cancer cells was less than nonmalignant cells (**Figures 5H–L**). In nonmalignant cells, such as immune cell types, the ferroptosis may be maintained such that normal level that ferroptosis drives active nearly or higher than ferroptosis suppresses active. However, tumor cells of these cancer types probably attempted to escape ferroptosis to some extent by successful growth and proliferation. Thus, the ferroptosis biological process is an important regulator in TME and is closely related to nonmalignant cells, in particular immune cell types.

One impressive observation is that the ferroptosis-associated signatures evaluated by RFL have potential effectiveness in cancer therapies. Our results in tissue level and single-cell level data showed that high ferroptosis level cases demonstrate both immunostimulatory and immunosuppressive phenotypes, as featured by high immune cell infiltrates and IC expression (**Figures 5**, **6A–C** and **Figure S3**), suggesting that these patients probably benefit from treatment based on the combination of immunotherapy and ferroptosis-correlated therapy. For example, a recent study established an effective strategy in LUAD to synergistically induce ferroptotic cancer cell death and reprogram the TME by transforming pro-tumor M2 macrophages to anti-tumor M1, decreasing the population of Tregs and inhibiting IC expression in CD8 T cells (36). The results of molecular characterization analyses also demonstrated that high RFL was associated with MSI/CIMP and inflammatory infiltrates subgroups (**Supplementary Figure 2**). MSI tumors are associated with good survival outcomes and response to IC inhibitor-related treatment in CRC (25, 26). Ferroptosis also regulates cell death to release a factor that mediates inflammatory and immune responses in TME (35). Additionally, ferroptosis levels were higher in response/sensitive tumor cases/cells under chemotherapy or radiotherapy treatment (**Figures 6D–F**). These findings are consistent with the studies in CESC that silencing components of ferroptosis signaling leads to radiotherapy resistance (40, 41). A prior study also showed that the chemosensitizing effects of Andrographis were mediated by activation of ferroptosis (42). Moreover, a recent study demonstrated that ferroptosis is cross-talk with immunocytes in GBM, which could offer a novel chemotherapy strategy (43). Hence, our systematic classification of ferroptosis levels in human cancers has crucial clinical implications and can help evaluate the clinical benefit of ferroptosis-targeted therapies combined with immunotherapy, chemotherapy, or radiotherapy.

Our study has some limitations. First, tumor tissue and single-cell samples from multiple datasets do not provide direct values for ferroptosis levels, such as MDA or lipid ROS level. We therefore had to indirectly infer the relative ferroptosis level through the RFL in cancer types, similar to previous pan-cancer studies (44, 45). Second, RFL-correlated genes identified by RFL need further validation and exploration to confirm their function and position in the ferroptosis signaling system. Nonetheless, we developed a simple and robust method to evaluate the ferroptosis level that considers the drive and suppress regulators in this study. The performance of this RFL was validated using independent datasets where ferroptosis status is known. This study provides a resource of ferroptosis to effectively predict clinical outcomes and identify new, probably RFL-correlated genes. We expect that our findings will lead to a deeper understanding of ferroptosis-related immunotherapy, chemotherapy, and radiotherapy.

## Data Availability Statement

The datasets presented in this study can be found in online repositories. The names of the repository/repositories and accession number(s) can be found in the article.

## Author Contributions

KC, LG, KW, and ZH designed the study. KC, LG, KW, BL, and QL performed bioinformatics analyses, proofread and visualization. KC, LG, KW, YW, and LH performed wet-lab experiments. QZ and BF provided conceptual advice. KC, LG, KW, and ZH wrote the manuscript with comments from all authors. All authors discussed the results. All authors listed have made a substantial, direct, and intellectual contribution to the work and approved it for publication.

## Funding

This work was supported by grants from the National Natural Science Foundation of China (82002550 and 82173063), the Wuxi Health Commission Project (Q202051), and the Wuxi Medical Innovation Team (CXTP003).

## Conflict of Interest

The authors declare that the research was conducted in the absence of any commercial or financial relationships that could be construed as a potential conflict of interest.

## Publisher’s Note

All claims expressed in this article are solely those of the authors and do not necessarily represent those of their affiliated organizations, or those of the publisher, the editors and the reviewers. Any product that may be evaluated in this article, or claim that may be made by its manufacturer, is not guaranteed or endorsed by the publisher.
