# Supplementary material for: Ferroptosis-Associated Molecular Features to Aid Patient Clinical Prognosis and Therapy Across Human Cancers
Source: Front Immunol. 2022 Jun 20;13:888757. doi: 10.3389/fimmu.2022.888757 (PMC9266629; doi:10.3389/fimmu.2022.888757)
Supplement: Supplementary file 1 [file DataSheet_1.pdf]

Ferroptosis-associated Molecular Features to Aid Patient Clinical Prognosis and Therapy across Human Cancers

Supplementary Figures

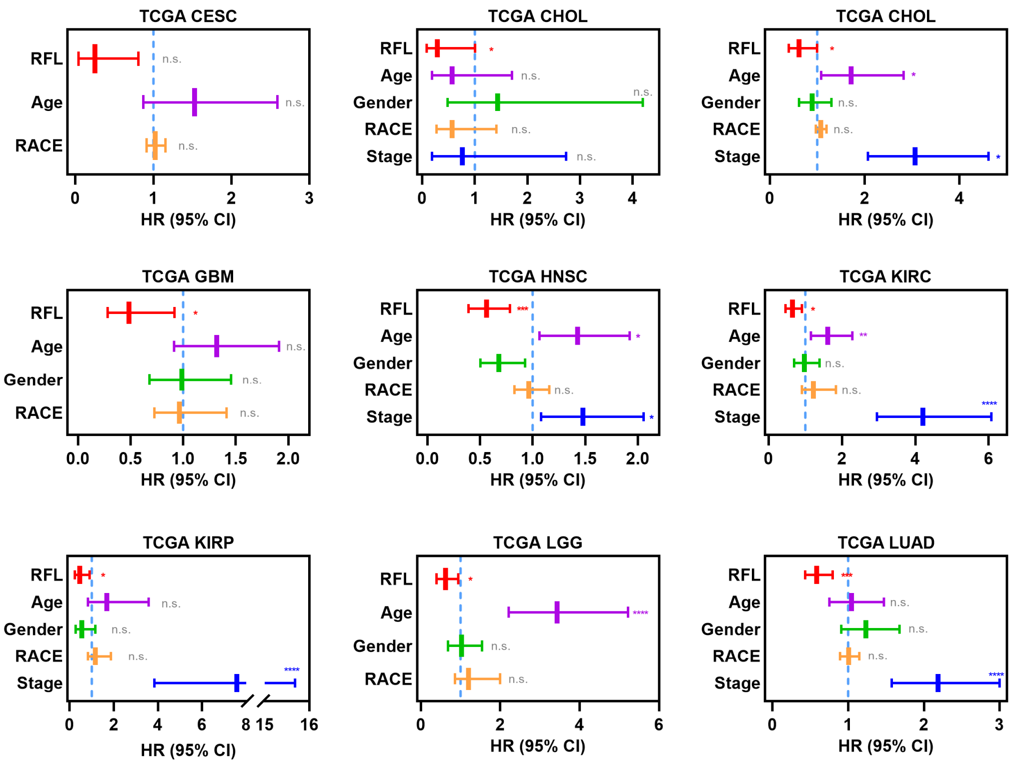

Figure. S1 Multivariate COX regression models showing the effect to OS of RFL, age, gender, race and TNM stage in TCGA cancer types.

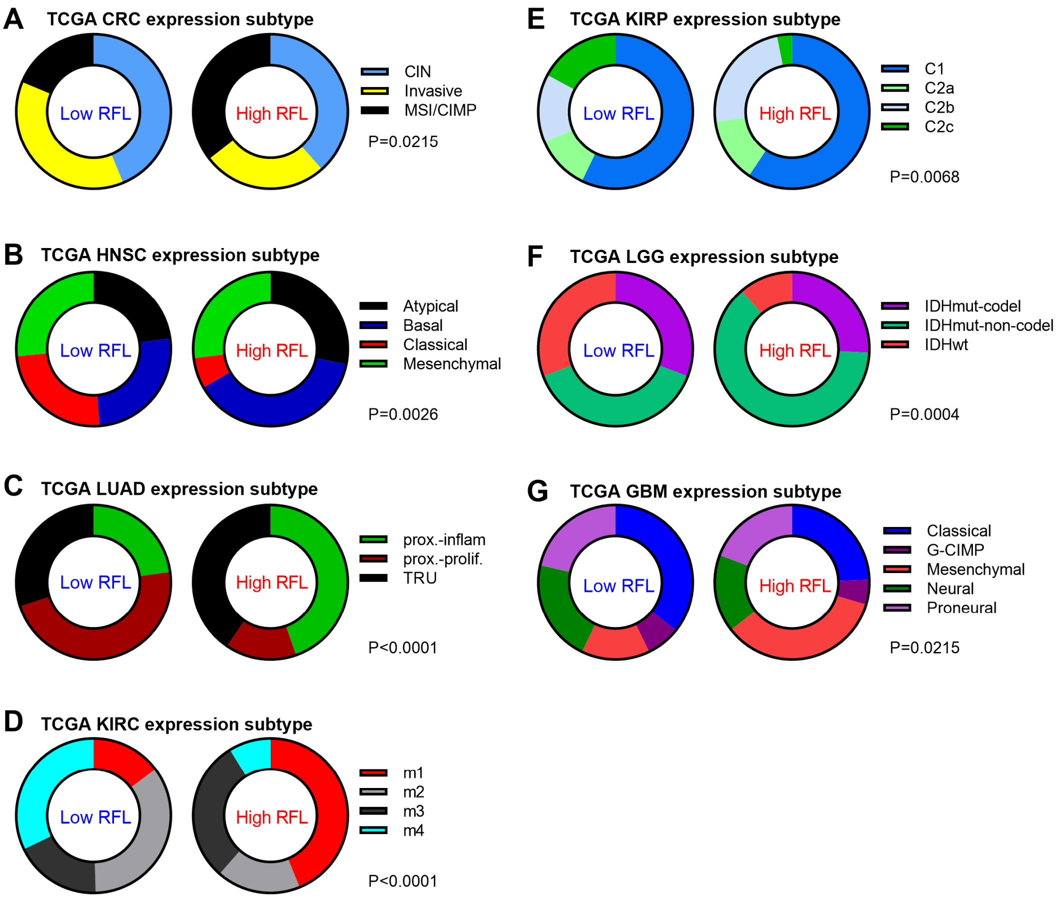

Figure. S2 Pie charts showing the distribution of TCGA CRC (**a**), HNSC (**b**), LUAD (**c**), KIRC (**d**), KIRP (**e**), LGG (**f**) and GBM (**g**) subtypes in high and low RFL groups.

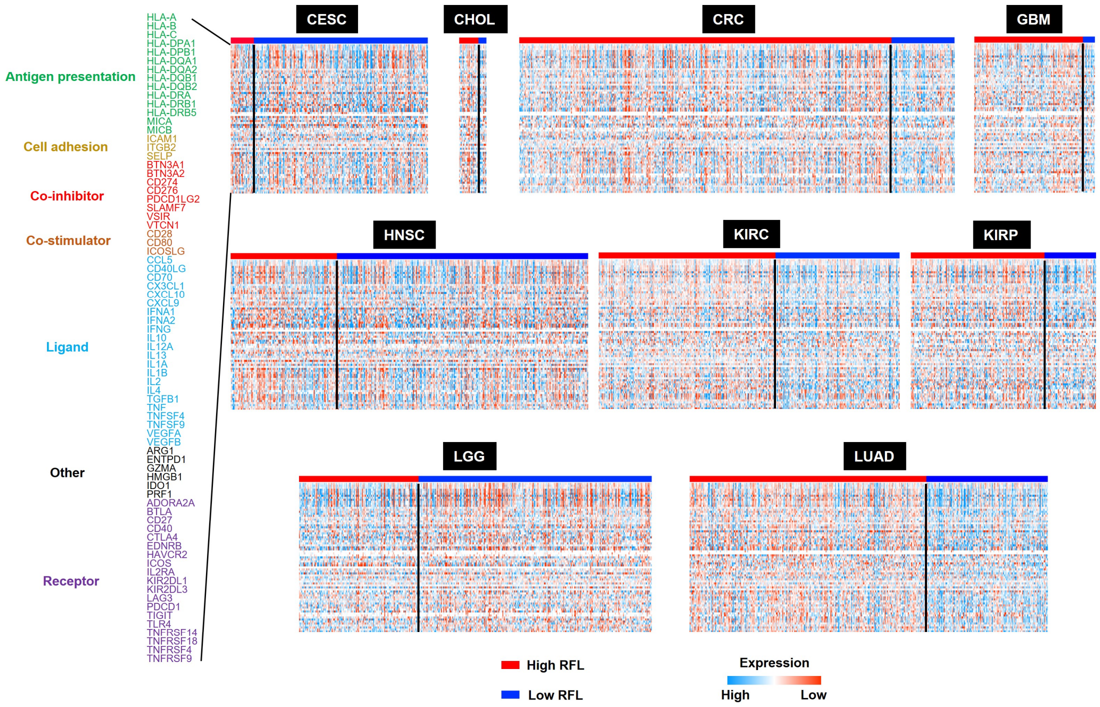

Figure. S3 Heatmap showing expressions for 75 immunomodulator genes group by high and low RFL groups in TCGA cancer types.
